# A pilot study on exertional tasks with physiological measures designed for the assessment of military concussion

**DOI:** 10.2217/cnc-2020-0018

**Published:** 2021-04-09

**Authors:** Julianna H Prim, Maria I Davila, Karen L McCulloch

**Affiliations:** 1 Department of Allied Health Sciences, Curriculum in Human Movement Science, University of North Carolina at Chapel Hill, Chapel Hill, NC 27599, USA; 2 Department of Psychiatry, University of North Carolina at Chapel Hill, Chapel Hill, NC 27599, USA; 3 Department of Allied Health Sciences, Division of Physical Therapy, School of Medicine, University of North Carolina at Chapel Hill, Chapel Hill, NC 27599, USA

**Keywords:** autonomic dysfunction, exercise intolerance, exertional tasks, heart rate variability, military concussion, primary care, return to activity

## Abstract

**Background:**

Guidelines for clinicians treating military concussion recommend exertional testing before return-to-duty, yet there is currently no standardized task or inclusion of an objective physiological measure like heart rate variability (HRV).

**Methodology & results:**

We pilot-tested two clinically feasible exertional tasks that include HRV measures and examined reliability of a commercially available heart rate monitor. Testing healthy participants confirmed that the 6-min step test and 2-min pushup test evoked the targeted physiological response, and the Polar H10 was reliable to the gold-standard electrocardiogram.

**Conclusion:**

Both tasks are brief assessments that can be implemented into primary care setting including the Polar H10 as an affordable way to access HRV. Additional research utilizing these tasks to evaluate concussion recovery can validate standardized exertional tasks for clinical use.

Concussion or mild traumatic brain injury (mTBI) is a prevalent injury in civilian, athletic and military populations. mTBI results in a variety of symptoms that limit activity. Both early return to normal activity and prolonged rest have been shown to increase symptom duration [1–3]. Clinicians commonly determine military duty readiness based on self-reported absence of symptoms and return to ‘normal’ performance on clinical assessments that may have ceiling effects for this population [4].

The Traumatic Brain Injury Center of Excellence (TBI CoE) has developed clinical recommendations for military primary care providers (PCP) for management of mTBI that outline a five-stage activity progression, similar to sports concussion consensus return-to-play recommendations [5–7]. TBI CoE guidelines recommend an exertional test before resumption of activity and again before return to duty. However, implementation of exertion testing is inconsistent, and there are no standardized exertional assessments that are feasible and validated for primary care.

Symptom self-report at rest serves as the primary measure that clinicians use to recommend return-to-activity [8], but physiological deficits may persist beyond symptom resolution [9,10]. Although concussive symptoms may have multiple causes, one contributor to impairment is autonomic nervous system (ANS) dysfunction [11,12]. The ANS drives communication between the brain and circulatory regulation that may be disrupted after mTBI [13,14]. Sympathetic and parasympathetic function is reflected in heart rate variability (HRV), which is the variation in time between successive heartbeats. HRV can serve as a proxy for ‘top-down’ integration of mechanisms that regulate peripheral physiology and can provide insight on stress and overall health [15]. One critical HRV component, respiratory sinus arrhythmia (RSA), or high frequency (HF), represents parasympathetic activity or vagal tone [15,16]. After concussion, it may be necessary to induce physical stress to observe subtle ANS and cardiovascular dysfunction [17].

The current gold standard for HRV measurement requires electrocardiogram (ECG) measurement [18] because the sequence of times between R-peaks provides a noninvasive measure of the neural regulation of the heart, but ECG is not always feasible. High-quality heart rate monitors (HRM) like the Polar H10 (Polar Electro Oy, Kempele, Finland) are a less expensive alternative with adequate reliability compared to ECG in a resting state, but lower reliability under higher exertion levels has been reported [19–21].

Exertional testing is a reasonable clinical approach to identify possible ANS dysfunction after concussion [22]. Standardized and validated exertional tasks for mTBI in acute and prolonged recovery are the Buffalo Concussion Treadmill Task (BCTT) [23] and a similar bike test [24], but time and equipment requirements make them more appropriate for rehabilitation settings. Military service members with concussion are typically followed by primary care for the first month postinjury. Although exertional testing [25] is encouraged to guide return to activity and duty, the required time, space, and equipment [26,27] are not available for most primary care providers. A field expedient test of exertion for PCPs guided by objective physiological measures could improve clinical feasibility and implementation.

The goals of the present study are to pilot test clinically feasible exertional tasks developed based on minimal time, space, equipment requirements and determine whether the objective physiological measure of HRV could be implemented during the testing protocols. We tested healthy adults, a majority of whom were service members (SMs), in two brief exertional tests: a stepping (STEP) and a pushup (PU) task that were easy to administer using readily available equipment and typical PCP exam room space [4,27]. We used a modified 6-min Chester Step Test, a graded step test validated for emergency service providers to quantify occupational aerobic capacity [28,29]. The step task progressively increases speed every 2 min, and similar to the BCTT [30] and HRV has been successfully collected during a stepping task [31]. The second exertional task was performance of pushups for 2 min. Pushups are part of the current Army Physical Fitness Test and an important training component for all military branches [32] with clear functional health relevance [33,34], but they have not been studied to test exertion after concussion. We hypothesized that HRV measurement before, during and after each task using clinically available equipment (Polar H10) would be reliable compared with the gold standard ECG (Faros 180, Mega Electronics Ltd., Pioneerinkatu, Finland) measurement. We also hypothesized that both tasks would be feasible for participants to complete and achieve targeted exertion levels and physiological responses.

## Methods

### Participants

All participants were healthy adults between the ages of 18 and 45 years who were active, exercising at least three times a week to be representative of the active duty military population. A majority of participants were affiliated with the US Marine Corps, serving in eastern North Carolina at a recruiting station. Exclusion criteria were any medical condition or injury that limited ability to perform a physical training session or moderate exertion of stepping or pushups for 10 min, history of moderate to severe TBI or self-reported concussion in the past year. Screening occurred during in-person briefings via review of inclusion/exclusion criteria and study procedures; individuals interested in participating could contact researchers.

### Testing procedures

**Figure 1.**
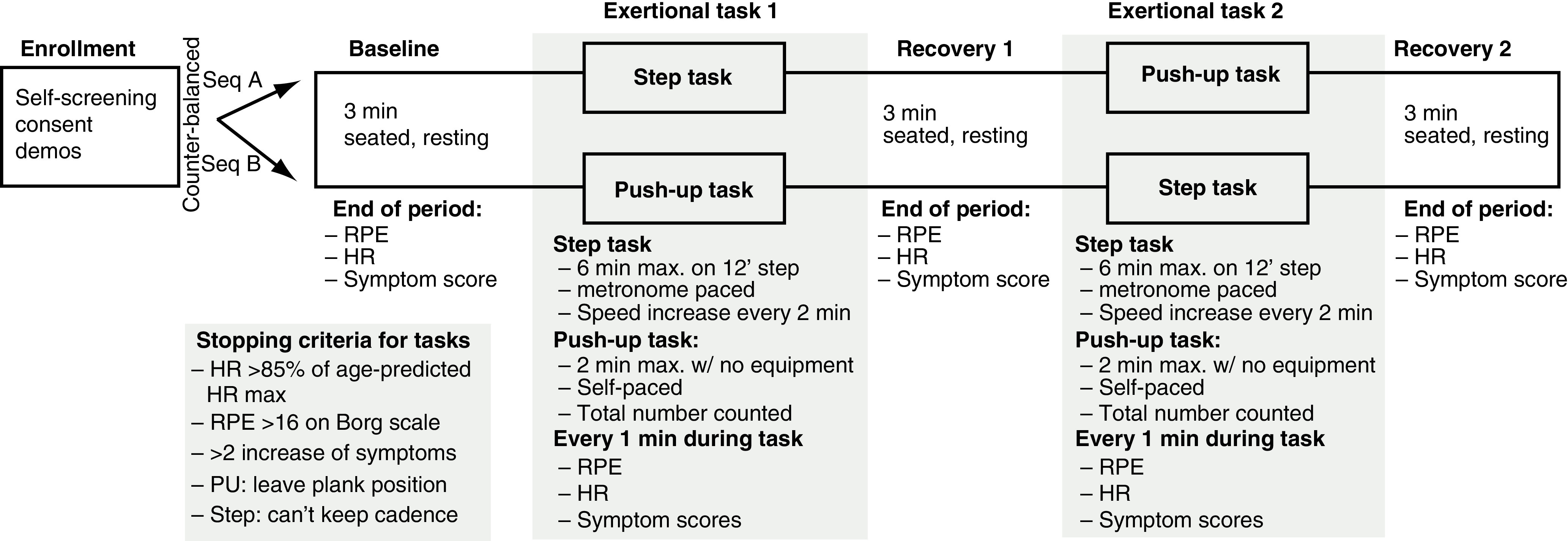
Layout of testing session. HR: Heart rate; PU: Pushup; RPE: Rate of perceived exertion.

Participants were seen for a single test session lasting approximately 45–60 min. We continuously recorded heart rate (HR) and interbeat intervals (IBIs) with two HRM (Polar H10 and Faros 180), during baseline, STEP and PU tasks (counterbalanced order) and recovery periods after each task (Figure 1). During exertion HR was monitored in real time via Bluetooth, and exertion level and concussive symptoms were surveyed each minute.

### Self-report measures

Participants completed a demographic questionnaire including questions on self-reported concussion history, military experience, current pain (0–10 scale), sleep (number of hours) and caffeine (number of drinks) within the past 24 h. Rate of perceived exertion (RPE) using the Borg Scale, a 6–20 scale reflecting subjective measure of workload, was used to document self-perceived exertion during exercise [35]. The presence or increase of symptoms was assessed using a 0–10 Likert scale focusing on headache, dizziness, nausea, light/sound sensitivity and fogginess, similar to the approach used in other exertional tests for concussion [23,36]. Throughout the session, we recorded verbal RPE and concussive symptom scores at the end of baseline (BL), rest following first test (R1), rest following second test (R2) and each minute during each exertional task, not expecting testing to cause symptom exacerbation.

### Heart rate recording

To examine the reliability of HRV measurement we employed the Faros 180 ECG monitor (Mega Electronics Ltd.) and the more clinically feasible Polar H10 monitor (Polar Electro Oy). Both monitors recorded HR and IBIs. Participants wore the Polar H10 around their chest and the Faros 180 was connected by three lead electrodes (right and left collarbone, left ribcage). The target range for heart rate (60–85% of predicted HRmax) was determined from the Fox and Haskell's equation (HRmax =220 -age[years]) [37]. This equation is a simple, commonly used part of the BCTT protocol [30] and has been recommended for use in military populations for graded exercise tests [38].

### Stopping criteria

We used BCTT guidelines for safety in our protocol to halt either test based on signs of excessive participant stress [39], including HR >85% of predicted HRmax, RPE >16, a reported increase >2 on the symptom scale over baseline values or the examiner perceived that testing was unsafe. Participants were also instructed that they could discontinue testing at any time if they deemed it necessary.

### STEP task

The step task was a maximum of 6 min in duration and required a 12-inch step and a smartphone metronome app. Every 2 min the stepping pace increased as a participant stepped up and down a 12-inch step (using preferred lead and trail legs) beginning at 80 bpm (20 steps/min), then 100 bpm (25 steps/min), and finally 120 bpm (30 steps/min). The test was discontinued based on safety stopping criteria or if the participant was unable to maintain the metronome pace.

### PU task

The PU task was a maximum of 2 min long. Participants were instructed to complete as many pushups as possible during the time duration without resting. This is especially relevant for military populations [33]. The test was discontinued for the stopping criteria or if the participant released from plank position to rest at any point. The total number of pushups was measured with a handheld counter by the examiner.

### Data processing & reduction

The Faros 180 recorded a complete ECG waveform at 1 kHz. IBIs, the time between consecutive heartbeats expressed in milliseconds, were derived from detected R peaks in ECG using the Cardio Peak-Valley Detector (CPVD) [40] to create the IBI event series. Polar H10 monitor automatically reduced the heart rate electrical signal to IBIs. Before analysis, both sequences of IBIs were synchronized automatically, then visually inspected to ensure proper alignment. Each aligned sequence was then transformed into a 2-Hz equally sampled time series by linear interpolation, extracting of HRV parameters while preventing the two series from becoming decoupled. The unedited IBI file was visually inspected and edited offline with CardioEdit software (developed in the Porges laboratory and implemented by researchers trained in the Porges laboratory).

HRV frequency components were calculated with CardioBatch software (Brain-Body Center, University of Illinois at Chicago), which implements the Porges–Bohrer method [41]. This method is neither moderated by respiration nor influenced by nonstationarity and reliably generates stronger effect sizes than other commonly used metrics of RSA [41]. Variables included average heart rate (i.e., normalized mean IBIs every 60 s), RSA defined by the frequencies of spontaneous breathing (0.12–0.4 Hz), low frequency (LF) HRV occurring within the frequencies of spontaneous vasomotor and blood pressure oscillations (0.06–0.10 Hz) and heart period (i.e., total HRV, mean IBIs) (Appendix 1).

### Data analysis

Means, standard deviations, medians, interquartile ranges and 95% CIs were calculated for all demographic and questionnaire data. Alpha was set *a priori* at α <0.05 for all statistical analyses. Normality was assessed for all dependent variables using the Shapiro–Wilk test. Only participants with complete data for both sensors were included in reliability analyses.

Reliability and accuracy of the Polar H10 IBIs compared with the Faros 180 IBIs were analyzed using Bland–Altman (B-A) plots and generalized estimating equations (GEE). Comparison of independent measurements was facilitated by visualizing the distribution between the mean measurement and the difference [42]. B-A plots, examined with SPSS statistical software (IBM SPSS Statistics, Version 26.0 [IBM Corp., NY, USA]), show agreement between two sensors, by plotting the mean between pair of measurements against its difference. Visual inspection of the B-A plots is used to identify systematic biases and possible outliers. Paired t-tests evaluated whether the differences between the signals were biased (i.e., one signal source generating longer or shorter values). B-A plots and the t-test were performed on IBIs collected from all participants during all tasks. Scatter plot and linear regression analyses were used to visualize and calculate the level of convergence between the Polar H10 and Faros 180. A strong correlation of threshold of R^2^ ≥0.9 of IBI time series was determined as a target representing strong agreement [43]. For each HRV component measure a GEE model was conducted using PROC GENMOD in SAS 9.5 (SAS Institute, NC, USA) to estimate group mean differences (95% CIs) for HR monitor methods (Polar H10 vs Faros) and session (BL, R1, R2, STEP, PU). This allowed evaluation of the effects of method of HR measurement on HRV components.

Clinical feasibility was assessed by participant completion of the tasks and the ability to record HRV at baseline, during exertion and throughout recovery. Physiological responses were assessed using HR and RPE measurements. HR was measured during both tasks with the exertional range (60–85% of age-predicted HRmax) as the primary target for a successful exertional task. Self-reported RPE between 12 and 16 (moderate exertion range) during the tasks was considered an appropriate physiological response.

## Results

### Participants

Fifteen healthy adults completed our testing protocol, 13 were active reservists for the US Marine Corps. Four of the Marine participants had a history of concussion, and nine had been deployed serving an average of 2.8 deployments (SD = 0.8). Full demographics are presented in Table 1.

**Table 1.** Demographic characteristics of all participants: military and health history for service members.

Demographic characteristic	n = 15
Age (years)	29.33 (6.36)
Sex Women Men	2 (13.3%) 13 (86.7%)
Race/ethnicity Caucasian Hispanic/Latino African–American Native American	7 (46.7%) 4 (26.7%) 2 (13.3%) 2 (13.3%)
Education High school Trade school Some college/associate's degree Bachelor's degree Advanced degree	1 (6.7%) 1 (6.7%) 8 (53.3%) 4 (26.7%) 1 (6.7%)
Military affiliation US Marine Corps None	13 (86.7%) 2 (13.3%)
**Military & health history**	**n = 13**
Time serving (years)	10.0 (5.5)
Military rank/pay grade E1–E5 E6–E9 O1–O3	4 (30.8%) 6 (46.1%) 3 (23.1%)
Deployment history Yes No	9 (69.2%) 4 (30.8%)
Concussion history Yes No	4 (30.8%) 9 (69.2%)
Behavioral health history Combat stress Posttraumatic stress Anxiety Depression	1 (7.7%) 2 (15.3%) 2 (15.3%) 1 (7.7%)
Caffeine (drinks/supplements in past 24 h)	1.9 (2.0)
Sleep (hours in past 24 h)	5.7 (1.2)

Values are n (%) or mean (SD).

### Reliability and accuracy of Polar H10

**Figure 2.**
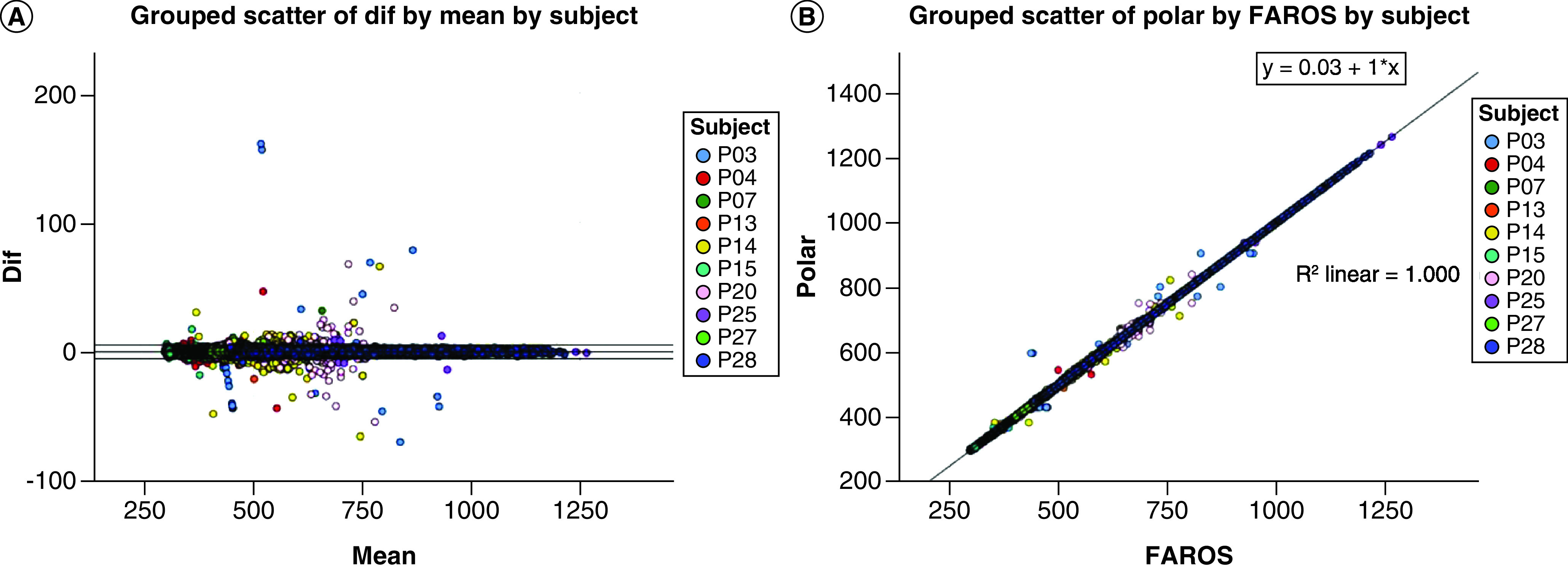
Bland-Altman and scatter plot for inter-beat interval from the Faros 180 electrocardiogram and Polar H10. **(A)** Plot of the IBI differences versus the means for the Faros 180 and Polar H10. Outer lines indicate the 95% confidence interval. **(B)** Scatter plot of the Faros versus Polar H10 IBIs with regression and R^2^. IBI: Inter-beat interval.

A subset of 10 participants had complete data for both the Polar H10 and the Faros 180. The Faros 180 was less able to detect HR peaks during pushups than the Polar H10, requiring more than 5% editing of total IBIs, beyond the recommended editing standard from HRV Task Force guidelines [18]. Therefore, the reliability analysis is based on data collected during STEP. Visual inspection of the B-A plot (Figure 2A) indicated excellent agreement and minimal bias between the sequential IBIs measured with Polar H10 and Faros 180. The mean of the differences between sensors was -0.0231 ms (SD = 3.197; t(23352) = -1.105 [95% CI: -0.064 to 0.017; p = 0.27]) with limits of agreement of -6.28 to 6.24 and no significant proportional bias (B = 0.0; t = 1.07; p = 0.28). The t-test results confirm that the pairs of sensors were measuring the same parameter. The mean of the differences was not significantly different from zero, indicating that there was no fixed sensor bias. The B-A plots suggested that error magnitude was driven by few participants and the IBI differences were closer to zero with longer IBIs (lower exertion). A scatterplot with regression analyses contrasting the sensor pair with linear regression of IBIs provide excellent fit to the IBI data with R^2^ of 1.00 driven by large amount of IBIs for the model of y = 0.03+x (Figure 2B).

**Figure 3.**
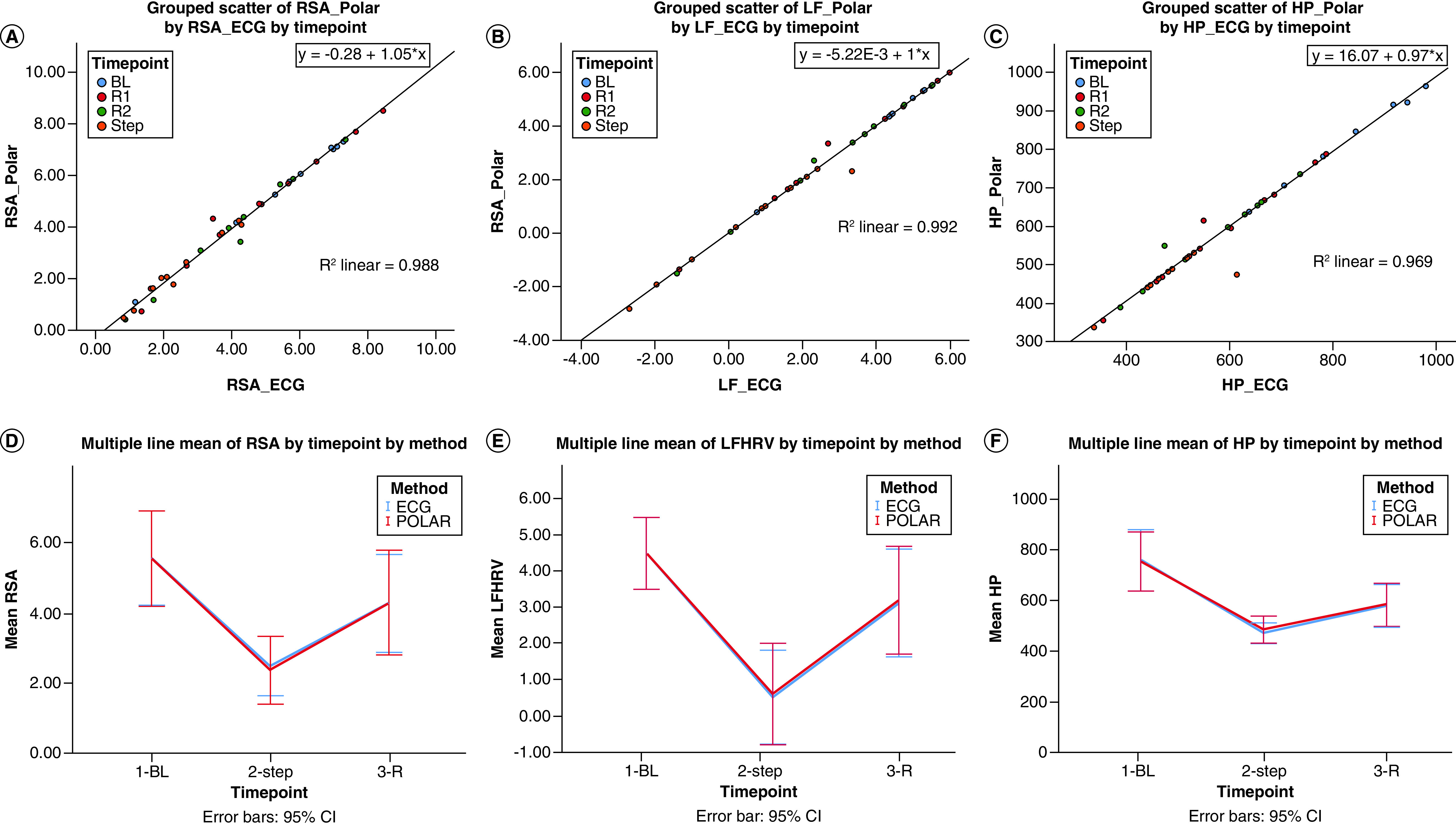
First row: Scatterplots between sensors for heart rate variability components, color-coded by time point with regression and R^2^. **(A)** RSA. **(B)** LF. **(C)** HP. D–F: Mean of HRV measures at baseline, exertion, and rest after exertion for Faros 180 electrocardiogram and Polar H10. **(D)** RSA. **(E)** LF. **(F)** HP. BL: Baseline; ECG: Faros 180; HP: Heart period; HRV: Heart rate variability; LF: Low-frequency; POLAR: Polar H10; R: Recovery period after STEP; RSA: Respiratory sinus arrhythmia; STEP: Step task. *Statistically significant at p < 0.05.

After HRV analyses were completed for the IBIs from both sensors, a scatterplot with regression analysis contrasting the derived HRV components from Polar H10 and Faros 180 confirmed excellent fit with R^2^ >0.95 (Figure 3A–C). GEE was used to demonstrate the sensitivity of both sensors regarding the change across time points in each HRV parameter (Table 2). For each HRV component (RSA, LFHRV, HP), sensor type was not a significant predictor, indicating that methods of HR recording were comparable (Figure 3D–F). STEP compared with other time points (BL, R1, R2) was a significant predictor of lower RSA, LFHRV and HP (Figure 3D–F).

**Table 2.** Generalized estimating equations analyses for the Polar H10 sensor compared with ECG and for each resting time point compared with exertion (STEP).

HRV component	Parameter		β	SE	95% CI	Z		Pr > |Z|
RSA	Sensor	ECG	0.086	0.048	-0.007	0.180	1.81	0.071
		POLAR	0.000	0.000	0.000	0.000	–	–
	Time point	BL	0.976	0.490	0.016	1.936	1.99	0.046*
		R1	2.048	0.428	1.208	2.887	4.78	<0.0001*
		R2	1.235	0.402	0.447	2.023	3.07	0.002*
		STEP	0.000	0.000	0.000	0.000	–	–
LFHRV	Sensor	ECG	0.026	0.025	-0.022	0.074	1.05	0.295
		POLAR	0.000	0.000	0.000	0.000	–	–
	Time point	BL	2.355	0.310	1.748	2.962	7.61	<0.0001*
		R1	2.347	0.356	1.649	3.044	6.59	<0.0001*
		R2	1.764	0.341	1.096	2.432	5.18	<0.0001*
		STEP	0.000	0.000	0.000	0.000	–	–
HP	Sensor	ECG	1.427	0.748	-0.039	2.892	1.91	0.063
		POLAR	0.000	0.000	0.000	0.000	–	–
	Time point	BL	284.089	36.562	212.430	355.749	7.77	<0.0001*
		R1	123.924	28.618	67.835	180.013	4.33	<0.0001*
		R2	103.123	28.129	47.991	158.256	3.67	0.0002*
		STEP	0.000	0.000	0.000	0.000	–	–

BL: Baseline; HP: Heart period; ECG: Faros 180; LFHRV: Low frequency heart rate variability; POLAR: Polar H10; R: Recovery period following step task; RSA: Respiratory sinus arrhythmia; STEP: Step task.

*Statistically significant at p < 0.05.

### Clinical & physiological feasibility

All 15 participants were able to complete both tasks as instructed without the examiner having to stop based on safety criteria. None of the participants reported symptom exacerbation during either task. HRV analysis was feasible for all of the phases (BL, ST, R1, PU, R2) based on IBI recordings from the commonly available Polar H10.

Both STEP and PU tasks evoked appropriate exertional physiological responses. All participants reached the exertional range (60–85% of age-predicted HRmax) during the 6-min step test and the 2-min push-ups. During STEP all participants reported a RPE between 12 and 16 at least once during the task. Fourteen of 15 participants reported a RPE in the exertional range (12–16) for PU.

## Discussion

The development of clinically feasible, standardized exertional tasks for PCPs to administer to SMs after an acute mTBI is an important step in the treatment and management of mTBI in accordance with TBI CoE recommendations. Our two exertional tasks were ecologically valid for SMs because they build on familiar tasks and are in use to test aerobic capacity and strength [29,33]. Our exertional task protocols appear feasible, induce adequate physiological responses and can be used to characterize HR recovery with an affordable HRM.

We found the Polar H10 recordings of beat-to-beat HR data for the exertional protocol collected through Bluetooth to be accurate and reliable compared with ECG recordings during the STEP, suggesting that it is a reasonable alternative for clinical use. Hernado *et al.* found HRM and ECG methods to be interchangeable when analyzing HRV at rest [19], but we also found excellent reliability and agreement indices of the HRV components between sensors under exertion. The use of clinically available HRM allows for straightforward administration and may increase utility for PCP.

Both the STEP and PU were feasible to perform during a PCP appointment, requiring less than 10 min, space consistent with a standard exam room and minimal or easily accessible equipment. Besides the Polar H10 HRM, the STEP requires a 12-inch step and metronome app, while the PU only requires a hand counter. Both tasks were easily conducted by one tester and could be completed in our largely military population without stopping for safety reasons. Previous studies have tested mTBI targeted assessments in healthy individuals before completing testing in a clinical cohort [44,45].

All participants demonstrated an appropriate HR exertion range during each task, indicating that these tasks were sufficiently challenging to cause the targeted physiological stress. RPE ratings also supported the use of the tasks, with only one participant rating below 12 on the RPE scale for pushups (participant stopped pushups at 30 s). Both exertional tasks are of greater difficulty than current commonly used concussion balance assessments, which may reduce test ceiling effects [4].

Exploratory analyses showed there were significant differences in HRV for the stepping task compared with the baseline and recovery time periods, indicating that HR monitors could sufficiently detect changes in all three HRV components induced by brief exertion. Similar to previous studies, we found a decrease in RSA when under exertion, consistent with the parasympathetic withdrawal that occurs upon initiation of exercise [46,47]. We also found a decrease in the LFHRV and HP [48,49].

As with any research, this study had limitations. First, we tested exertional tasks in a healthy population, therefore, future studies need to investigate tolerance of these exertional tasks for individuals who have sustained a concussion to characterize possible HRV impairments compared with healthy controls. However, our majority military study cohort supports the feasibility of tasks and physiological response in our target population. Second, comparisons between Polar H10 and Faros 180 did not include the pushup task because of concerns with peak detection in the Faros 180 leading to overediting. This finding further supports the use of the Polar H10 because it may be more reliable at recording valid IBI data during pushups and similar exercises, as well as being commercially available. In addition, only ten participants were used in reliability calculations due to initial Bluetooth technical difficulties leading to missing data. Yet with more than 15 min of IBI data for each participant, the sample size reflects previous studies, and the total number of IBIs supports adequate power in reliability analyses [40]. Furthermore, inclusion of a commercial Bluetooth dongle resolved connectivity dropout with Polar H10 and improvement to our data platform allows for continual saving to minimize any data loss due to technical issues.

Feasibility testing in a healthy, largely military population allowed improvements to the protocol for future studies. For instance, the duration of the baseline and recovery time periods was increased from 3 to 5 min. Although 3-min recovery between tasks was sufficient for these healthy participants to return to RPE of 6, we expect that individuals with concussion may need longer to recover. Additionally, we will add measures of medicine and alcohol intake, which can also influence HRV values.

## Conclusion

The implementation of standardized exertional tasks that includes an objective physiological measure may improve the standard of care for military mTBI. Monitoring symptoms, RPE, and HR during exertional tasks assesses physiological recovery and informs activity recommendations [35]. The treatment and management of concussion remains a priority for TBI CoE and the armed forces. Further research is needed to determine the utility of such measures with acute concussion in order to facilitate clinical implementation of exertional testing by PCPs.

## Future perspective

Research about concussion and the ability to begin activity after 24–48 h of rest after injury is increasing. Military PCPs are in a position to offer guidance about progressive activity by considering more than self-reported symptoms if they have validated performance-based tests that are feasible in the office setting. Wearable sensors are increasingly used by civilian and military populations and may provide additional evidence for activity progression after concussion.

**Summary points**

- The Department of Defense Traumatic Brain Injury Center of Excellence guidelines for Primary Care Providers treating concussion recommends brief exertional testing before return to duty, yet there is currently no standardized task validated for that purpose.
- Heart rate variability (HRV) is an objective measure of autonomic nervous system activity and may be useful in assessing physiological impairments after concussion.
- Comparing reliability of an affordable commercially available heart rate monitor (Polar H10) to the gold standard electrocardiogram (ECG) under exertional conditions and verifying the sensitivity of HRV changes with new test protocols was a necessary step toward development of new clinical tests.
- With input from military medical providers, two brief exertion tests were developed that could be easily administered in a primary care office environment requiring minimal space, equipment and time: a 6-min metronome-paced step test and a 2-min pushup test.
- A sample of largely military healthy participants successfully completed both tasks, with a 3-min baseline and a 3-min rest after each task with the order counterbalanced.
- Both tasks evoked the targeted physiological response of 60–85% of predicted heart rate maximum and moderate rate of perceived exertion.
- The reliability of the Polar H10 was better than the ECG during the pushup task and comparable during the step task, favoring the use of the Polar H10 as an affordable and easier to use device to capture HRV.
- Both the step and pushup task are brief, clinically feasible assessments that could be used in primary care practice as measures of recovery.
- A standardized test incorporating HRV measurement could be used with self-report of symptoms to aid clinicians in prescribing activity and managing recovery.
